# The Outcome of Papillary Thyroid Cancer Associated with Graves' Disease: A Case Control Study

**DOI:** 10.1155/2018/8253094

**Published:** 2018-05-08

**Authors:** Riju Menon, C. Gopalakrishnan Nair, Misha Babu, Pradeep Jacob, G. Praveen Krishna

**Affiliations:** ^1^Endocrine Surgery Division, Amrita Institute of Medical Sciences, Kochi, Kerala 682041, India; ^2^Department of General Surgery, Endocrine Surgery Division, Amrita Institute of Medical Sciences, Kochi, Kerala 682041, India

## Abstract

**Introduction:**

Thyroidectomy is now a less popular therapeutic option for Graves' disease. The frequency of thyroid nodule and the cancer risk of these nodules accompanying Graves' disease are controversial. The outcome of thyroid cancers coexisting with Graves' disease is debated.

**Study Design:**

Designed as retrospective case control study of papillary thyroid cancers associated with Graves' disease and those with euthyroid background. Pathological characteristics and outcome of papillary thyroid cancers in the two groups were compared.

**Results:**

The tumour characteristics did not differ significantly in the groups. The patients were followed for a mean period of 77.32 months and found significant incidences of disease progression in patients with papillary thyroid cancer associated with Graves' disease (*p* = 0.034; OR 2.747, CI 1.078–7.004). Disease progression as new distant metastases mostly in skeletal locations was high in this group compared to euthyroid group (*p* = 0.027; OR 4.121, CI 1.008–15.600). There was higher incidence of cumulative metastatic diseases in papillary thyroid cancer associated with Graves' disease.

**Conclusion:**

Papillary thyroid cancers associated with Graves' disease show aggressive biological behaviour and favoured site of distant metastases was osseous locations. Early diagnosis by routine screening of Graves' disease patients with ultrasound imaging and aspiration studies is recommended.

## 1. Introduction

Traditionally thyroid cancers were considered uncommon in patients with hyperthyroidism. One of the earliest reports of thyroid cancer in exophthalmic goitre was recorded in 1948 by Pemberton and Black [1]. Subsequently many anecdotal case reports and case series appeared in literature establishing this rare association. But the actual incidence of thyroid cancer in hyperthyroidism is still uncertain since the studies were mostly in subset of Graves' disease (GD) who underwent surgical treatment.

Diffuse thyromegaly is the characteristic feature of GD and nodules are rarely seen. In a multicentre retrospective analysis of 557 consecutive patients who underwent thyroidectomy for Graves' disease, the overall prevalence of nodule was 25.1% [2]. Nodule prevalence was high when ultrasound imaging (US) was employed for screening and Cantalamessa et al. detected 33.6% prevalence of nodules among 315 patients with GD [3]. A collective review showed nodule prevalence of 10% to 15% among GD patients and the malignancy rate of 2.3% to 45.8% (mean 16.9%) [4]. These figures are higher than approximately 5% nodule prevalence and 5% reported cancer risk of thyroid nodule occurring in general population [4, 5]. Patients with Graves' disease and coexisting thyroid nodules were almost 5 times more likely to have thyroid carcinoma than those without nodules [6].

The biological behaviour of thyroid cancers in GD is debated and researchers like Hales et al. did not find any difference in the histological characteristics and outcome of such tumours [7, 8]. On the other hand, Ozaki et al. recorded frequent locoregional and distant metastases in Differentiated Thyroid Cancer (DTC) patients with GD [9]. Pellegriti et al. [10] compared long-term outcome of 21 thyroid cancers associated with Graves' disease to that of euthyroid thyroid cancers matched for age, sex, and tumour size. They concluded that the cumulative risk for recurrent or progressive distant metastases was approximately threefold higher in Graves' patients than in euthyroid patients. Similar observations were made by other authors also [11].

This retrospective case control study was done on thyroid cancer patients who underwent treatment in our Institution during 2005 to 2012. All patients with GD in the present series had thyroid cancers of papillary histology and so control group of similar histology was generated. The aim of the study was to find long-term outcome of PTC associated with Graves' disease. The secondary aim was to find the differences in tumour characteristics of such cancers. We hypothesized that long-term outcome of papillary thyroid cancers associated with Graves' was poor compared to that of euthyroid counterparts.

## 2. Study Design

Case records of patients who were operated on for Differentiated Thyroid Cancer (DTC) during the study time were reviewed. Patients with histological diagnosis of papillary thyroid cancer (PTC) were included in the study. Diagnosis of Graves' disease (GD) was based on clinical and biochemical features of hyperthyroidism, technetium^99^ scintiscan, and histological features. Preoperative preparations included thionamides and beta-blockers but supersaturated iodine preparations were not used. Indications for thyroidectomy for hyperthyroidism were large volume goitres (>90 ml), suspicious cytology, grade II or III active orbitopathy, and patient's preference. All patients underwent high resolution ultrasound imaging and aspiration cytology from the nodules and suspicious lymph nodes.

Primary surgical treatment was Total Thyroidectomy (TT) when preoperative diagnosis was available. Therapeutic central compartment lymph node dissection was always done but prophylactic dissection was not routine. Selective neck dissection of lateral neck nodes clearing nodal stations in Levels II (A&B), III, IV and V was done when metastasis was detected.

The histological analysis was based on WHO guidelines [12]. Histological variants such as tall cell, columnar cell, and hobnail variant were grouped together as aggressive variants for analysis [13]. Risk stratification was done based on AJCC Cancer Staging Manual, 7th edition [14].

All patients underwent endogenously stimulated whole body I^131^ scan [WBS] with 3 millicuries [mCi] of I^131^ 3 weeks after thyroidectomy. Remnant ablation was done in patients with tumour size >2 cm, presence of extrathyroid invasion, angioinvasion, aggressive histology, or residual locoregional disease.

Patients were reviewed on six-month interval with complete clinical examination, estimation of stimulated thyroglobulin, and thyroglobulin antibodies along with neck ultrasound imaging. I^131^ WBS and FDG Pet were done based on clinical and laboratory evaluation. In the context of elevated thyroglobulin (>10 ng/ml) with negative ultrasound localization, I^131^ WBS was done to screen for regional or distant metastases. When I^131^ WBS was negative and serum thyroglobulin was above 50 ng/ml FDG Pet was done. The diagnosis of distant metastasis was made when iodine or FDG avid lesion was detected with elevated serum thyroglobulin levels.

## 3. Outcome

Outcome was measured based on ATA guidelines 2015 [13].

1. Excellent response: no clinical, biochemical, or structural evidence of disease
2. Biochemical incomplete response: abnormal thyroglobulin or antithyroglobulin antibody levels in the absence of localizable disease
3. Structural incomplete response: persistent or newly identified locoregional or distant metastases
4. Indeterminate response: nonspecific biochemical or structural findings.

Disease progression was defined as development of new regional or distant metastasis during follow-up period. For study purpose, patients who had PTC with GD were grouped as PTC-GD group.

## 4. Statistics

The cohort was grouped based on the presence of coexisting Graves' disease and group to group analysis of differences in prevalence of demographic and tumour features was done. The variables used for outcome analysis included age in years, male gender, tumour size, presence of extrathyroid invasion, multifocality, vascular invasion, lymph node spread, and Graves' disease. For the comparison of categorical variables among the different groups, Chi-square test was used (*p* = 0.05). The one-way analysis of variance (ANOVA) is used to determine whether there are any statistically significant differences between the means. Data analysis was performed using the SPSS (Chicago, IL) statistical package.

## 5. Results

During study period, 308 patients underwent primary surgery for DTC which included 28 Follicular Thyroid Cancers and 12 Hurthle cell cancers. Also during the study period, 165 patients with hyperthyroidism were operated on of which 102 were diagnosed as GD. There were no thyroid cancers detected in autonomous hyperthyroidism but 29 (18%) had nonoccult papillary cancers associated with GD. All these patients had preoperative confirmed or suspicious cytological diagnosis of papillary thyroid cancer.

We analyzed the data of 268 patients with PTC with a mean age of 41.1 years (SD 13.582) and 32.5% were above 45 years. The cohort included 197 females (73.5%). One patient of euthyroid group had skeletal metastasis at the time of diagnosis. Primary surgery included TT alone for 139 (51.9%), TT with level VI dissection for 81 (39.2%), TT with unilateral neck node dissection for 44 (16.4%), and TT with bilateral neck node dissection for 4 (1.5%) patients. Histology of these patients showed PTC of classic variant (*n*: 186, 69.4%), infiltrative follicular variants (*n*: 71; 26.5%), and aggressive variants (*n*: 11; 4%).

Group to group analysis of the clinicopathological features is shown in Table 1. Tumour size was significantly small in PTC-GD patients compared to euthyroid counterparts. Risk stratification showed low incidence of higher stages in GD group (Table 2).

Pretreatment I^131^ WBS showed 32 incidental locoregional residual diseases and 5 incidental distant metastases. One patient of PTC-GD group had skeletal and pulmonary metastases and 4 of euthyroid group had pulmonary metastases. Seventy-nine patients (29.5%) did not receive I^131^ ablation and the remaining received remnant ablation. Forty-five (16.8%) required additional doses of I^131^ metastatic radioiodine therapy. The mean amount of I^131^ used in PTC-GD group was higher than that of euthyroid group (145.4 mCi (SD 131.28) versus 72.3 mCi (SD 80.335); *p* = 0.001). But the euthyroid group required 21 resurgical procedures during follow-up period whereas the PTC-GD group did not have any (*p* = 0.082).

All patients were followed for mean duration of 77.3 months (24–144, median-74). Of the cohort disease progression was found in 20 (7.4%) patients. This included 5 [17%] of PTC-GD group and 15 [6.3%] of euthyroid group (*p* = 0.034; OR 2.747, CI 1.078–7.004). Disease progression as new distant metastases was in 3 (10.3%) of PTC-GD group and 6 (2.5%) of euthyroid group (*p* = 0.027; OR 4.121, CI 1.008–15.600) (Table 3).

At the end of follow-up period, 243 (91%) patients had excellent response. Four (1.5%) patients died of disease related complications and one in euthyroid group died of coronary artery disease. Twenty (7.5%) had structural incomplete response and 3 had biochemical incomplete response. Excellent response was in 91.6% (*n*: 219) of patients of euthyroid group and 82.8% (*n*: 24), *p* = 0.116, of patients in the PTC-GD group. Details of final outcome are noted in Table 4. Osseous metastases were seen in 4 (13%) of patients in PTC-GD group and 8 (3%) of euthyroid group. Mean duration of disease before thyroidectomy was not different in the group with distant metastasis and those without (12.48 months versus 11.45; *p* = 0.256).

## 6. Discussion

All histological types of DTCs were found associated with GD but we observed papillary histology as the exclusive subtype. The high incidence of cancer in GD patients is biased by referral pattern and routine institutional treatment protocol of GD. Radioiodine ablation is the most favoured definitive procedure for GD.

Generally features like male gender, age over 45 years, larger tumour size, extrathyroid invasion, nodal metastases, and follicular histology were found to influence the outcome of DTC negatively [15, 16]. But in the present series tumours in PTC-GD subgroup were smaller in size and did not differ in the distribution of aggressive histological features. The pTNM staging did not show stage IV diseases in PTC-GD group. Belfiore and colleagues found that tumours associated with GD were often multifocal, larger in size, and locally invasive and are metastatic to lymph nodes or to distant sites compared to patients with autonomous thyroid nodules and attributed the aggressiveness to circulating TSAb [17]. The present study did not include TSAb as a variable in the analysis since many patients were referred from peripheral centres after long-term treatment with thionamides. We conclude that the initial clinical and histological features PTC associated with GD are not different from those of euthyroid cancers.

There was no difference in radioiodine avidity in the GD group and so management protocol was the same for both groups. The total amount of I^131^ used in GD group was considerably high despite the similar histological characteristics probably due to frequent incidences of disease progression. The euthyroid cancers had higher frequency of locoregional disease requiring surgical procedures.

The overall Disease-Free Survival (DFS) of the cohort was 91% and is at par with many recorded series [18]. DFS of the PTC-GD group was low but the difference did not attain a statistical significance. Pellegriti et al. found 57.1% disease-free patients in DTC-GD group compared to 87.1% in euthyroid patients after a median follow-up of 165.6 months [19]. But Yano and colleagues found no difference in the clinical and histological features of 154 PTC in GD patients compared to euthyroid controls and course of the disease did not show any difference in persistence of the disease or cancer-related death [20]. The tumour size of the series was small (median 0.9 cm; 0.1–70.0) and majority were of T1 category. We found a considerable high rate of disease progression in PTC-GD group compared to euthyroid counterparts despite similar tumour characteristics. At the end of follow-up period, the cumulative metastatic disease was significantly high in PTC-GD group.

Distant metastases occur in less than 10% of patients with papillary and follicular thyroid carcinoma but represent the most frequent cause of thyroid cancer-related death. Lungs are the most favoured site for metastases in papillary carcinoma and account for 70% of all distant metastases [16, 21]. But we observed osseous metastases more frequent in PTC-GD group. The overall reported incidence of bone metastasis from papillary thyroid carcinoma has ranged from 1.7% to 7% [22, 23]. The rate of osseous metastases was 13.6% in PTC-GD group which were either isolated or along with pulmonary metastases. Ren and colleagues found young age as a significant predictor of distant metastases [24].

The study was done in a significant number of papillary thyroid cancers in Graves' disease but carry all weakness of retrospective analysis. The serum level of TSAbs was not included in the analysis since most of the patients received thionamides for considerable period prior to seeking treatment in the Institution.

## 7. Conclusion

Papillary carcinomas associated with Graves' disease show aggressive behaviour even when tumour characteristics are favourable. Routine US imaging with aspiration helps early diagnosis of DTC in Graves' disease. Disease progression as osseous metastases is more frequent in PTC associated with Graves' disease. These patients warrant more aggressive treatment and vigilant follow-up compared to euthyroid counterparts.

## Figures and Tables

**Table 1:** Clinicopathological feature.

Features	PTC-GD group	Euthyroid group	*p* value
Mean age in years (SD)	40.59 (10.872)	40.87 (13.584)	0.603
Male gender	10 (34.5%)	58 (24%)	0.156
Tumour size (mm)	24.69 (11.619)	33.41 (21.569)	0.033
	Median-23	Median 26.8	
Classic variant	21 (74%)	165 (69%)	0.123
Aggressive variants	3 (10.3%)	9 (3.8%)	0.128
Multifocality	12 (41.4%)	64 (26.8%)	0.099
Extra thyroid invasion	6 (20.7%)	50 (21%)	0.941
Angioinvasion	9 (31%)	78 (32.6%)	0.522
Level-6 nodes	7 (24%)	78 (32.6%)	0.240
Lateral neck	6 (20.7%)	46 (19%)	0.807

Total	29	239	

**Table 2:** PTNM staging.

	Stage I	Stage II	Stage III	Stage IV a	Stage IV b	Stage IV c
PTC-GD group	20 (69%)	5 (17%)	4 (14%)	0	0	0
Euthyroid group	167 (70%)	27 (11%)	30 (13%)	12 (5%)	0	3 (1%)

**Table 3:** Details of disease progression.

Features	PTC-GD group	Euthyroid group	*p* value
Mortality	1 (3.4%)	3 (1.3%)	0.358
Distant metastases	3 (10.3%)	6 (2.5%)	0.027
Locoregional recurrence	1 (3.4%)	4 (1.7%)	0.246
TENIS^*∗*^	0	3 (1.3%)	0.534

Total	29	239	

^*∗*^Thyroglobulin elevated negative iodine scan.

**Table 4:** Details of adverse outcome.

Site of metastases	PTC-GD	Euthyroid
Bone	2 (6.8%)	1 (0.4%)
Lung	0	2 (0.8%)
Bone + lung	2 (6.8%)	4 (1.6%)
Local recurrence + lung	0	3 (1.3%)
Local recurrence + lung + bone	0	3 (1.3%)
Local recurrence	1 (3.4%)	4 (1.7%)
